# Axillary vein thrombosis induced by an increasingly popular oscillating dumbbell exercise device: a case report

**DOI:** 10.1186/s13019-015-0264-3

**Published:** 2015-05-20

**Authors:** Hani Shennib, Kelli Hickle, Bradley Bowles

**Affiliations:** 1 Arizona Heart Hospital, 1930 East Thomas Road, Phoenix, AZ 85016 USA; 2 University of Arizona, College of Medicine–Phoenix, 550 East Van Buren Street, Phoenix, AZ 85004 USA

**Keywords:** Upper extremity, Venous thrombosis, Axillary vein thrombosis, Thrombolytic therapy mechanical thrombectomy, Paget-Schroetter syndrome, Thoracic outlet syndrome, Shake Weight, Dumbbell

## Abstract

A 53 year-old male presented with a one-day history of a swollen arm and dull, aching pain in the right upper extremity. The patient reported commencing exercising daily over the prior week with a modified, oscillating dumbbell; commonly referred to as a Shake Weight. Imaging revealed an occlusive thrombus in the right axillary, proximal brachial and basilic veins. The patient was treated with a 24-hour tPA infusion followed by mechanical thrombectomy, balloon angioplasty, and stent placement for a residual thrombus and stenosis. The patient was discharged the following day on warfarin and aspirin. This is the first report of effort-induced thrombosis of the upper extremity following the use of a modified, oscillating dumbbell. Due to the growing popularity of modified dumbbells and the possible risk for axillary vein thrombosis, consideration should be made to caution consumers of this potential complication.

**Electronic supplementary material:**

The online version of this article (doi:10.1186/s13019-015-0264-3) contains supplementary material, which is available to authorized users.

## Background

Upper extremity deep venous thrombosis (UEDVT) is a thrombus within the subclavian, axillary or brachial vein. UEDVT is much less common than it’s lower extremity counterpart, but is associated with similar complications including: post-thrombotic syndrome, recurrent thrombosis, and rarely, pulmonary embolism. Thrombosis of the upper extremity is divided into primary and secondary causes; primary UEDVT occurs spontaneously in the setting of strenuous upper extremity effort or in the absence of known thrombotic risk factors. Secondary UEDVT is related to a known risk factor such as malignancy or central venous catheters [1,2].

The thoracic outlet is bound by the clavicle and subclavius muscle anteriorly, the scalenus anticus muscle laterally, the first rib posterior-inferiorly and the costoclavicular ligament medially. Compression of the neurovascular bundle within the thoracic outlet is termed thoracic outlet syndrome (TOS) and is present in approximately 60% of patients with UEDVT [1]. Effort-induced thrombosis, or Paget-Schroetter syndrome, is a clinical manifestation of TOS caused by distortion and narrowing of the vein within the tunnel by repetitive upper extremity exercise. Historically, Paget-Schroetter syndrome has been associated with unusual pursuits or occupations including: golf, tennis, baseball, football, weight lifting, painting, etc. [3] A literature review of recent case reports reveals UEDVT associated with baseball, surfing, and weight lifting [4-6]. Per Urschel and Razzuk, the preferred treatment for Paget-Schroetter syndrome is thrombolytic therapy followed by prompt resection of the first rib for neurovascular decompression [3]. However, surgery is not without its risks (e.g. pneumothorax, nerve injury, etc.) and no randomized controlled trials have been performed to investigate the optimal treatment of UEDVT [1].

This is the first report of the development of effort-induced thrombosis of the upper extremity following the use of an increasingly popular, modified, oscillating dumbbell.

## Case presentation

**Figure 1:**
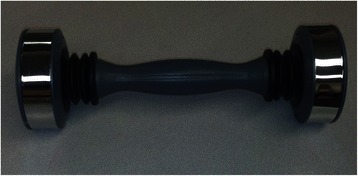
Oscillating dumbbell exercise device. A photograph of the patient’s modified, oscillating dumbbell, which he commenced exercise with one week prior to presentation with an upper extremity deep vein thrombosis.

This is a 53 year-old African American male, with no significant past medical history who presented with a one-day history of right arm swelling. He woke up with a swollen arm and dull, aching pain in the right upper extremity. Our patient, a retired office manager, reported exercising daily over the prior week with a new “vibrating device” (Figure 1). Patient denies any past interventions to the right upper extremity.

### Clinical findings

Patient denied: known malignancy, personal or family history of DVT, trauma, intravenous therapy, and drug abuse. Physical examination was unremarkable except for extensive right upper extremity swelling.

### Timeline

See Additional file 1.

### Diagnostic assessment

Laboratory testing revealed CBC, CMP, PT/INR and aPTT all within normal limits.

**Figure 2:**
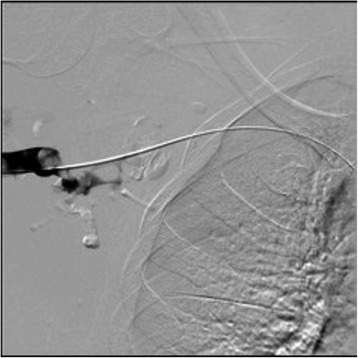
Venogram 1. Venography confirmed extensive occlusion of the right axillary, proximal brachial and basilic veins.

A venous doppler study revealed an occlusive thrombus in the right axillary, proximal brachial and basilic veins. Access via a forearm vein was achieved and venography confirmed extensive occlusion of the right axillary, proximal brachial and basilic veins (Figure 2).

### Therapeutic intervention

**Figure 3:**
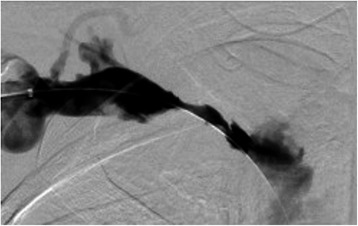
Venogram 2. Venography following 24 hours of tPA infusion revealed small residual thrombus and 50% stricture in the right axillary vein.

**Figure 4:**
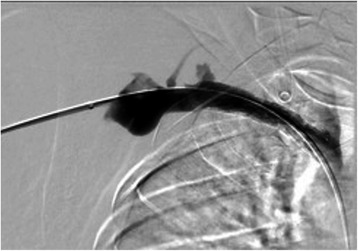
Venogram 3. Completion angiography showed complete resolution of the stricture and thrombus following mechanical thrombectomy, balloon angioplasty, and stent placement.

An infusion catheter was positioned to allow tPA infusion of the thrombus throughout its entire length over the ensuing 24 hours. The following day, a venogram revealed the presence of a small residual thrombus and a 50% stricture in the right axillary vein (Figure 3). Mechanical thrombectomy using an angiojet device, followed by balloon angioplasty and deployment of a 12 × 6 mm stent resolved the residual thrombosis and stenosis. Completion angiography showed complete resolution of the stricture and thrombus (Figure 4).

### Follow-up and outcomes

The patient was discharged the following day on warfarin and aspirin. There were no adverse or unanticipated events.

## Discussion

Our patient exercised regularly for years and began using the device only one week prior to developing an UEDVT. The device is a modified dumbbell with a centralized spring and weight attached to either end. When shaken, the weight bounces off either end creating self-perpetuated oscillations and increased resistance. The resistance leads to sustained thoracic wall and arm muscle contraction, purportedly increasing the effects of exercise by forcing the muscles to work harder. Fitness IQ, the company that makes the original device, the Shake Weight, has termed this technology “dynamic inertia” [7]. In 2010, Shake Weight sales had reached $40 million with the expectation of selling 10 million more units [8].

In the absence of any other etiology for thoracic outlet syndrome or other precipitating factors, we hypothesize that our patient developed an axillary vein thrombosis as a result of the recently initiated exercise with the device. We hypothesize the mechanism of injury to the axillary vein when such devices are used is repeated compression and traumatization to the vein by the contracted muscles that form the boundaries of thoracic outlet, thereby initiating clot formation. In this case, first rib resection was not pursued due to the belief that it was initiation with the device alone that caused the thrombosis. However, should the patient experience a recurrence, first rib resection would be a viable treatment option.

## Conclusions

This is the first report of the development of effort-induced thrombosis of the upper extremity following the use of an increasingly popular, modified, oscillating dumbbell. This association has never been reported and is worth further monitoring. Due to the growing popularity of modified dumbbells and the possible risk for axillary vein thrombosis, consideration should be made to caution consumers of this potential complication. Pharmacological and mechanical thrombolysis is effective in resolving the axillary vein thrombosis and should be considered in healthy, athletic individuals.

## Consent

Informed consent was obtained from the patient for publication of this Case report and any accompanying images. A copy of the written consent is available for review by the Editor-in-Chief of this journal.

## Additional file

Additional file 1:
**Timeline of interventions and outcomes.**

## Abbreviations

UEDVT: Upper extremity deep vein thrombosis
TOS: Thoracic outlet syndrome
tPA: Tissue plasminogen activator
