# Supplementary figures and images for: Axillary vein thrombosis induced by an increasingly popular oscillating dumbbell exercise device: a case report
Source: J Cardiothorac Surg. 2015 May 20;10:73. doi: 10.1186/s13019-015-0264-3 (PMC4446062; doi:10.1186/s13019-015-0264-3)

## Timeline:

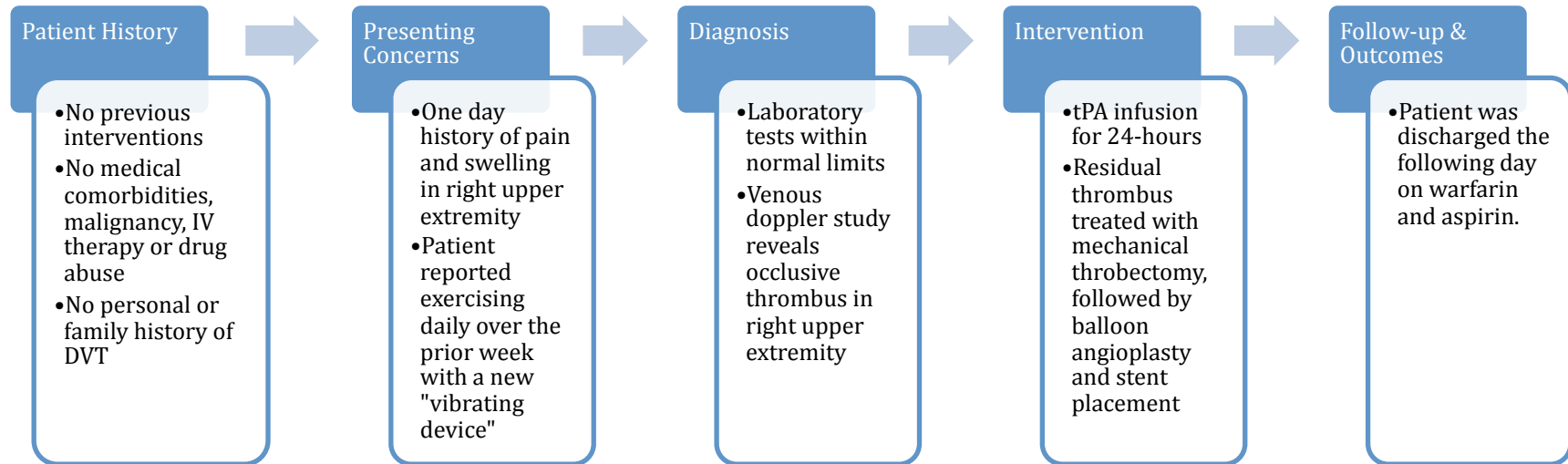

Supplement: Additional file 1: — Timeline of interventions and outcomes. [file 13019_2015_264_MOESM1_ESM.pdf]
